# Closed-loop therapy: recent advancements and potential predictors of glycemic outcomes

**DOI:** 10.1080/17425247.2025.2492363

**Published:** 2025-04-18

**Authors:** Chloë Royston, Roman Hovorka, Charlotte K Boughton

**Affiliations:** Institute of Metabolic Science-Metabolic Research Laboratories, University of Cambridge, Cambridge, UK

**Keywords:** Automated insulin delivery, hybrid closed-loop, fully closed-loop, type 1 diabetes

## Abstract

**Introduction:**

Hybrid closed-loop systems have become the standard of care for managing type 1 diabetes (T1D). Both clinical trials and real-world data have demonstrated that these systems improve glycemic control without increasing the risk of hypoglycemia, while also reducing the overall burden of T1D management. A systematic literature search was conducted using PubMed for studies including individuals with T1D that were published until the end of 2024.

**Areas covered:**

In this review, we summarize the safety and efficacy of currently available hybrid closed-loop systems, drawing from key clinical trials and real-world data analyses. We also highlight recent advancements in closed-loop systems, discuss their limitations and barriers to access, and explore future directions for automated insulin delivery. Finally, we explore potential predictors of outcomes for people with T1D to better understand why some individuals respond better to closed-loop systems than others.

**Expert opinion:**

Closed-loop systems are advancing rapidly, with a growing focus on enhancing automation through fully closed-loop systems to improve glycemic control and further reduce the burden of management. Identifying the predictors that influence how individuals respond to closed-loop therapy will enable these systems to be optimized. It is crucial to ensure widespread and equitable access to this advanced technology.

## 1.0 Introduction

Type 1 diabetes (T1D) is a chronic autoimmune disease requiring constant monitoring and management to prevent both immediate risks and long-term complications, placing a significant self-care burden on individuals and healthcare systems. Its incidence continues to rise; a meta-analysis by D’Souza et al. found increased cases in children from 2019 to 2021, and coupled with increasing life-expectancy, global prevalence is expected to double by 2040 [1, 2]. Over the past decade, there have been significant advancements in the management of T1D, particularly with the development of continuous glucose monitors (CGM), insulin pumps, and more recently hybrid closed-loop systems. These systems comprise of an insulin pump, CGM and algorithm housed either on the insulin pump or a compatible mobile device. Hybrid closed-loop systems have become the standard of clinical care to manage T1D as they increase time in range (70 to 180 mg/dL) without increasing hypoglycemia while also reducing the burden of care [3–5]. However, despite this, only about one-quarter of individuals with T1D in the United States and Europe achieve the treatment targets recommended by the American Diabetes Association (ADA) and the European Association for the Study of Diabetes (EASD) (HbA1c of <53 mmol/mol or 7% without significant hypoglycemia) [6–8]. This is likely due in part to the lack of widespread adoption of new technologies; in the UK, fewer than 20% of people with T1D use insulin pumps, and even fewer use closed-loop systems [7] and in the US 58% of individuals with T1D use insulin pumps. [9] Although usage continues to increase, this highlights the need both to improve access to technology and enhance closed-loop therapies.

Currently available closed-loop systems improve time in range, but individual responses to the algorithms vary. Identifying predictors of better glycemic outcomes would be helpful to better understand why this occurs.

A systematic literature search was conducted using PubMed for studies published up until the end of 2024, and included additional articles known to authors. Relevant articles were identified using predefined search terms related to closed-loop systems and automated insulin delivery in individuals with T1D.

In this review, we summarize the key clinical evidence from randomized control trials, single-arm trials, and real-world analyses of the available hybrid closed-loop systems across different age groups. We also highlight advancements in closed-loop therapies, their limitations, and potential future directions. Finally, we examine predictors of outcomes for users of these systems.

Management of T1D in pregnant women is not in the scope of this review but there are studies and reviews that address this population [10–12]. Similarly, type 2 diabetes (T2D) is not within the scope of this review, but other reviews and articles discuss technological advancements relevant to T2D specifically [13–15].

## 2.0 Benefits of closed-loop therapy

### 2.1 Overview

Hybrid closed-loop therapy has been shown to be safe and effective across all ages with T1D. The key randomized clinical trials organized by age group are shown in Table 1. Table 2 contains single-arm trials for systems and age groups that do not have evidence from randomized clinical trials. Mean improvements in time in range (TIR, 70 – 180 mg/dL) ranged from 7 to 15 percentage points, depending on the age group, the comparator therapy, and baseline time in range / HbA1c. This was achieved either with a significant decrease in hypoglycemia or without a significant increase in time below range (TBR, <70 mg/dL). In addition to improving glycemic control, closed-loop systems reduce fear of hypoglycemia, have shown the potential to improve sleep, and ultimately reduce the burden of diabetes management for individuals with T1D and their caregivers [16–19]. These systems not only improve glycemic outcomes, but also improve quality of life.

While closed-loop systems offer clinical value for everyone with T1D, different populations face unique challenges and risks of hypoglycemia or hyperglycemia, so it is important to assess safety and efficacy of closed-loop systems across all age groups.

### 2.2 Young children

Young children are fully dependent on their caregivers for diabetes management. This population faces unique challenges due to unpredictable food consumption, physical activity, and behaviors, as well as hormonal and developmental changes. They also experience greater variability in insulin requirements compared to adults [20]. Young children may struggle to recognize or communicate symptoms of low blood glucose effectively [21]. This combined with the increased variability means caregivers frequently experience increased fear of hypoglycemia. These factors add to the complexity of T1D management and mean there is a high burden of care on caregivers.

There is good clinical evidence showing the benefit of hybrid closed-loop systems in this population. Randomized clinical trials have demonstrated the safety and efficacy of CamAPS FX and Tandem Control-IQ in very young children, showing an increased TIR of approximately 10 percentage points, compared to standard therapy, without a significant increase in hypoglycemia (Table 1) [22, 23]. Single-arm clinical trials demonstrate improved glycemic control without an increase in TBR, compared to baseline, with Omnipod 5 and Medtronic 780G (Table 2) [24, 25]. Currently CamAPS FX, Omnipod 5 and Tandem Control-IQ are the only approved systems for very young children below 6 years old (Table 4).

### 2.3 Adolescents

Adolescents with T1D face unique physiological, psychosocial and behavioral challenges that often result in increased HbA1c levels and suboptimal glucose outcomes [26–29]. Physiological changes during puberty make glucose management more difficult, while the typical pressures of adolescence, such as fitting in socially, while also trying to balance diabetes management add another layer of complexity [27, 30–32]. While some studies indicate there is no definitive evidence that adolescents with T1D have an increased risk of mental health conditions compared to their peers, most literature suggests they do experience a significant mental burden [33, 34]. Therefore, in addition to often more challenging diabetes management, adolescents are particularly vulnerable, frequently neglecting their diabetes which further reduces the likelihood of meeting the recommended targets [33]. Fear of hypoglycemia can cause some individuals to deliberately raise their blood glucose levels, particularly before physical activities or sleep [27, 32]. Similarly, disordered eating behaviors often involve intentionally increasing blood glucose levels. This is relatively common in this population, with one study reporting that 42% of adolescents and young adults engage in these behaviors [35]. Additionally, this age group is often transitioning to greater independence in their diabetes management, which can further impact glycemic outcomes [36].

Closed-loop systems have been shown to improve glycemic outcomes in this population without increasing hypoglycemia, while also reducing the mental burden and improving diabetes specific quality of life [16]. Randomized clinical trials with CamAPS FX, Diabeloop DBL4K, Do-It-Yourself (DIY) AndroidAPS, iLet, Medtronic 780G and Tandem Control-IQ showed an improvement of between 7 and 13 percentage points compared to the control therapy (either multiple daily injection (MDI), insulin pump therapy, sensor-augmented pump therapy or sensor-augmented pump therapy with predictive low glucose suspend (PLGS)) (Table 1) [37–43]. This was achieved without a significant increase in TBR. Omnipod 5 was also proven to be safe in this population, as demonstrated in a single-arm clinical trial comparing its use to baseline (Table 2) [44].

Up to 50% of adolescents omit or delay boluses for meals or snacks [45]. Using simplified meal announcements, rather than precise carbohydrate counting is one option to mitigate this; a feasibility study using three pre-set meal announcements with Medtronic 780G showed that adolescents were still able to meet international glycemic targets using this approach rather than accurate carbohydrate counting [46]. A study of 16 adolescents using an open source fully closed-loop system (with no bolusing for meals or snacks) also met the international glycemic control targets with a mean TIR of 81% [45]. These studies suggest that modified closed-loop systems could help to address the issue of adolescents forgetting to bolus for meals.

### 2.4 Adults

Young adults face similar issues to adolescents as they often move to new environments away from support systems, start new jobs and navigate the usual challenges associated with young adulthood while also trying to balance T1D management [47]. Older adults also face specific challenges; as age and duration of diabetes increases, so does the likelihood of complications and hypoglycemia unawareness [48–50]. This means optimal glycemic outcomes are vital to prevent further complications, but preventing hypoglycemia is also important.

Randomized controlled trials of Control-IQ, CamAPS FX, DBLG1, iLet, Medtronic 780G and Omnipod 5, show an average increase in TIR of 9 to 18 percentage points, compared to control therapy, without significant increase in TBR (Table 1) [39–42, 51–53]. Hybrid closed-loop therapies have also been shown to improve TIR specifically in older adults (≥ 60 years old), without increasing the risk of hypoglycemia while also reducing burden of management [54–56].

## 3.0 Currently available closed-loop therapies

**Figure 2:**
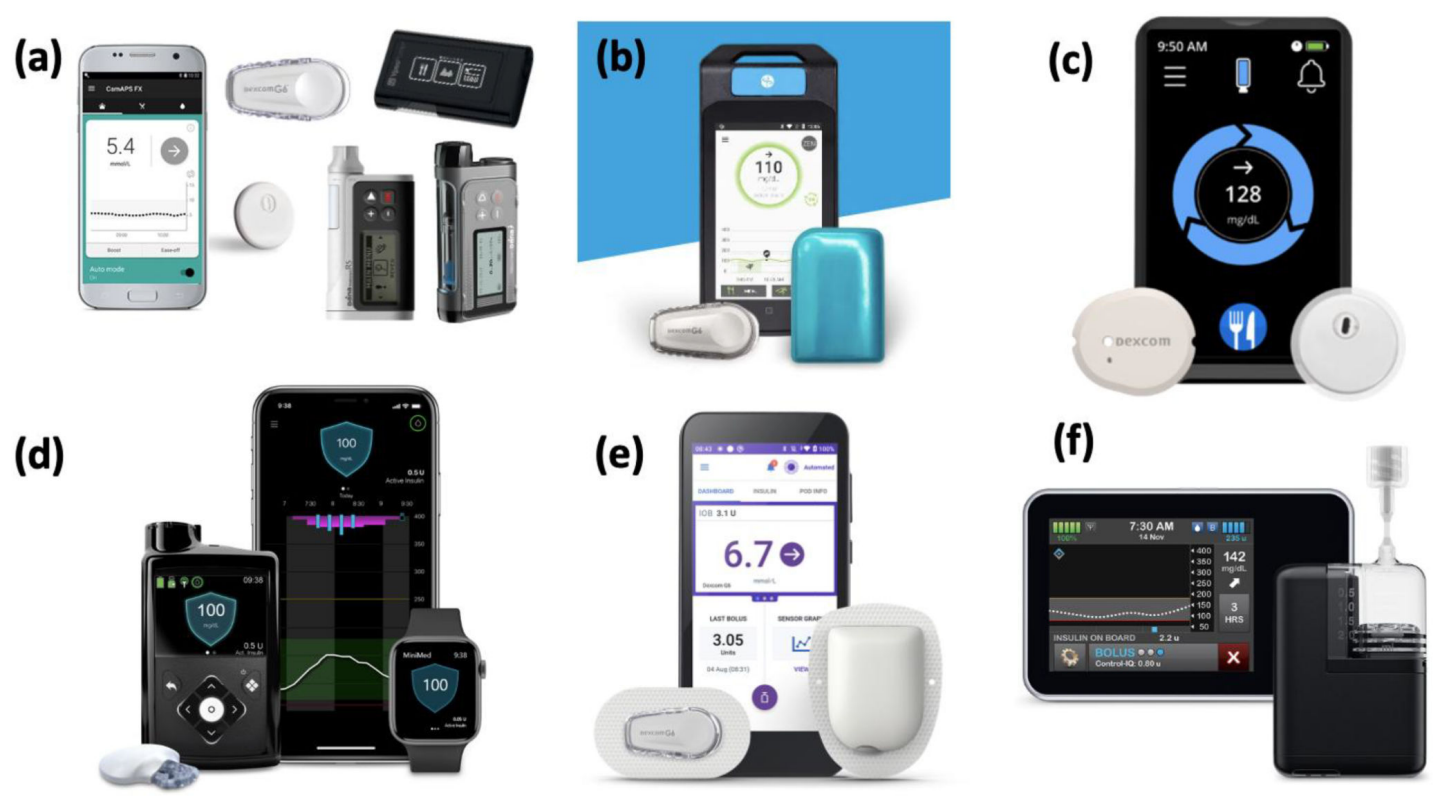
Commercially available automated insulin delivery systems (a) CamAPS FX (b) Diabeloop (c) iLet (d) Medtronic 780G (e) Insulet Omnipod 5 (f) Tandem t:slim and Mobi Control-IQ

Currently, there are six clinically approved closed-loop insulin delivery systems (Figure 2), one approved system that is not yet commercially available (based on the “do it yourself” Loop), and several non-approved “do it yourself” systems. Closed-loop systems consist of a distinct algorithm sometimes on a mobile device and operates with a compatible insulin pumps and continuous glucose monitor (CGM). The details of each approved system in clinical use are presented in Table 4. Each algorithm aims to keep glucose levels within a target range by adjusting the amount of insulin delivered every 5 to 10 minutes based on real-time glucose levels from a CGM, and a variety of factors described in Table 4. Each algorithm is unique but algorithms are usually based on model predictive control (MPC), proportional integral derivative (PID) or fuzzy logic (FL) controllers. These systems are all hybrid which means they still require the user to input and bolus insulin for meals. One system (the iLet) does not require carbohydrate counting but still requires meal announcements [3, 5]. Open-source systems can be used with or without meal announcements provided certain settings are enabled. Target glucose levels can be adjusted to suit the user, and some systems can be set to deliver more or less insulin for specific situations, such as illness or exercise. While closed-loop systems have been proven to be safe and effective in clinical trials it is important to assess their efficacy in the real-world. The key real-world studies for the different systems can be found in Table 3. Person-reported outcomes (PROs) are outside the scope of this review but further information can be found in the review by Kadiyala et al. [57].

### 3.1 CamAPS FX

CamAPS FX is an interoperable hybrid closed-loop app that uses Dexcom G6 or Freestyle Libre 3 CGM, mylife YpsoPump or DANA Diabecare RS or DANA-I insulin pump, and a smartphone which hosts the algorithm. The algorithm initially requires the user’s weight and total daily dose, and then calculates insulin sensitivity and active insulin time, which are automatically adjusted as necessary. The algorithm adapts to the user according to total daily dose, patterns from the day and from different mealtimes. A default glucose value of 104 mg/dL is targeted but this can be adjusted to any value between 80 and 200 mg/dL. CamAPS FX is one of only two systems to be approved for use in very young children (Table 4).

CamAPS FX has been proven to be safe and effective across all ages (≥ 1 years old) in randomized clinical trials (Table 1) [22, 37, 53] and in the real-world. Mean TIR was 73% in the real-world retrospective data analysis (Table 3), and was similar to the clinical trial outcomes across the different age groups (Table 1) [22, 58]. Median TBR was 2.3% (Table 3) [58].

### 3.2 Diabeloop DBLG1

Diabeloop DBLG1 is a hybrid closed-loop system available in Europe. It consists of the DBLG1 algorithm housed on a handset, Kaleido insulin pump and Dexcom G6 CGM. The algorithm is self-learning and inspired by the MPC controller. It is based on a physiological framework and consists of both short and long term learning algorithms as well as an algorithm for meal management. It uses the CGM reading, combined with these algorithms to adjust settings and insulin doses (either via basal delivery or bolus) every 5 minutes. Target glucose levels and sensitivity can be adjusted via the use of the “aggressiveness”, “zen”, or “activity” modes. It is approved for use in individuals with T1D who are ≥ 18 years old (Table 4).

Both clinical trials and real-world data demonstrated the safety and efficacy of this system in adults (≥ 18 years old). The median TIR was 69% in the clinical trial while TBR was 2.0% (Table 1) [51]. Real-world data showed a TIR of 72% and TBR of 0.9% (Table 3) [59].

### 3.3 iLet

The iLet bionic pancreas consists of the iLet insulin pump hosting the algorithm and either the Dexcom G6 or G7 CGM. The algorithm has three integrated algorithms – one for basal insulin, another for bolus corrections and a third meal awareness algorithm. This system does not require carbohydrate counting but does require meal announcements using one of three sizing options. This system is only available in the United States and is approved for use in people with T1D ≥ 6 years old (Table 4).

Clinical trials have demonstrated the safety and efficacy of this system (Table 1), however there are currently no published real-world data analyses available [40].

### 3.4 Medtronic 780G

The Medtronic 780G advanced hybrid closed-loop consisting of an algorithm on the 780G insulin pump and either the Guardian 3 (requires calibration) or Guardian 4 (no calibration) CGM. The algorithm has adaptive learning and is based on a PID controller with insulin feedback. It is approved by the FDA and is CE marked for individuals with T1D aged ≥ 7 years old.

The clinical trials demonstrated safety and efficacy in users aged 7-80 years old (Table 1) and safety in users aged 2-6 years old (Table 2) [25, 41, 43]. There is also an extensive retrospective data analysis of over 100,000 individuals with T1D using the Medtronic 780G aged ≥2 years old. The mean TIR was 72% while TBR was 2% (Table 3) [60].

### 3.5 Omnipod 5

Omnipod 5 is a hybrid closed-loop system that consists of the disposable Omnipod 5 patch pump (pod), the algorithm, controller (or compatible mobile device) and a CGM (Dexcom G6, Freestyle Libre 2 Plus or Dexcom G7 in the United States). The algorithm is based on the MPC controller. Basal insulin rates are calculated for each pod based on the recent total daily dose. Every 5 minutes, the CGM readings are used to predict glucose values for the next 60 minutes and then insulin microboluses are delivered as required to maintain the glucose level within the set target. Omnipod 5 is one of only two systems to be approved for use in very young children (Table 4).

In a randomized parallel trial in ≥18-year-olds, TIR was 44% in the control group compared to 61% with Omnipod 5 [52]. A single-arm trial also showed this pattern in children (TIR increased from 52% at baseline to 68% in the trial and 66% during the 3-month extension) and adolescents and adults (TIR increased from 64% at baseline to 74% after the study and 73% during the extension) [61].

A large 12-month retrospective real-world analysis was completed for individuals ≥2 years old on Omnipod 5. The 22,162 users aged 2 to 17 years old had a median TIR of 61% and TBR of 1.2% while those aged 18 and older (N=47,740) had a TIR of 66% and TBR of 0.9% (Table 3) [62].

### 3.6 Tandem Control-IQ

The Tandem Control-IQ system consists of the Tandem tslim:X2 insulin pump or Mobi insulin pump (US only) and the Dexcom G6, G7 or Freestyle Libre 2 Plus CGM. The Mobi insulin pump is controlled via an app on a compatible mobile device. The algorithm is based on an MPC controller but uses preprogramed basal rates and insulin sensitivity factors. There is no adaptive learning (Table 4).

While this system is not yet approved for very young children, a clinical trial with 2 to 6 year olds with T1D demonstrated safety and efficacy in this population [23]. A retrospective analysis of 12 months of data showed the system was also safe and effective in the real-world setting; the median TIR was 74% (64% at baseline) while TBR was approximately 1% (Table 3) [63].

### 3.7 Open source “DIY” systems

The open source closed-loop systems are highly customizable and consist of an algorithm housed on a mobile device, an insulin pump, a CGM and sometimes a device (Rileylink) linking the insulin pump to the mobile device. These systems must be set up by users themselves so may not be usable by everyone with T1D. Compatible insulin pumps are Accu-Check Combo / Insight, DanaR, DanaRS, Dana-I, Diaconn G8, EOPatch2, Omnipod Eros / DASH, Medtrum Nano /300U, Medtronic 515/715/522/722, Medtronic 523/723 (firmware 2.4 or lower), Medtronic Worldwide Veo 554/754 (firmware 2.6A or lower), or Medtronic Canadian/Australian Veo 554/754 (firmware 2.7A or lower) while compatible CGMs are Dexcom G5/G6/G7/ One/One+, Libre 1 (third party transmitter), European Libre 2, 2 Plus, or Minimed Enlite with the corresponding insulin pump.

The main DIY systems are Loop, OpenAPS, AndroidAPS and Trio. All systems predict glucose levels based on current CGM readings, insulin on board, carbohydrates on board and user defined insulin sensitivity and insulin to carbohydrate ratios. The amount of insulin delivered via basal rate is adjusted or micro boluses are delivered to keep glucose levels within the specified target. OpenAPS or Trio are based on the Oref1 algorithm which also incorporates dynamic insulin sensitivity and an algorithm that deals with unannounced meals. AndroidAPS also allows micro bolusing, uses dynamic sensitivity and can deal with unannounced meals. Therefore, these systems can be used with or without meal announcements depending on the settings. Loop, on the other hand, includes “glucose momentum” and “retrospective correction” settings, which are short-term adaptations designed to help the system manage rapid changes in glucose levels. These features also make the system more responsive by allowing it to adapt to unseen factors.

These systems are not approved by the FDA or CE marked, however clinical trials demonstrate their safety and efficacy (Table 1) [39, 45]. This is extended to the real-world where a prospective study (with 558 people aged 1 to 71 years old) was completed. Mean TIR was 73% while median TBR was 2.8%, compared to 67% and 3% at baseline (Table 3) [64]. The randomized clinical trial in 16 – 70 year olds had similar results with a baseline TIR of 65% increasing to 75% without a significant increase in TBR (Table 1) [39].

Tidepool Loop, is an FDA approved version of the open-source automatic insulin delivery (AID) algorithm, Loop. Tidepool Loop is an interoperable automated glycemic controller that links an interoperable alternate controller enabled (ACE) pump and integrated continuous glucose monitoring system (iCGM). It is not yet in clinical use as there are no commercially available insulin pumps compatible with Tidepool Loop [65].

### 3.8 All systems

Most systems report similar TIR with acceptable TBR in the real-world setting. It is important to note that direct comparisons between systems are not possible, as participants in each trial and real-world populations have different baseline characteristics and environments.

## 4.0 Potential predictors of glucose outcomes with closed-loop

Many factors influence glucose homeostasis, some of which may be used to predict potential glucose outcomes like TIR when using a closed-loop system. However, these factors are often interlinked, making it challenging to assess the predictive capability of each one individually.

**Figure 3:**
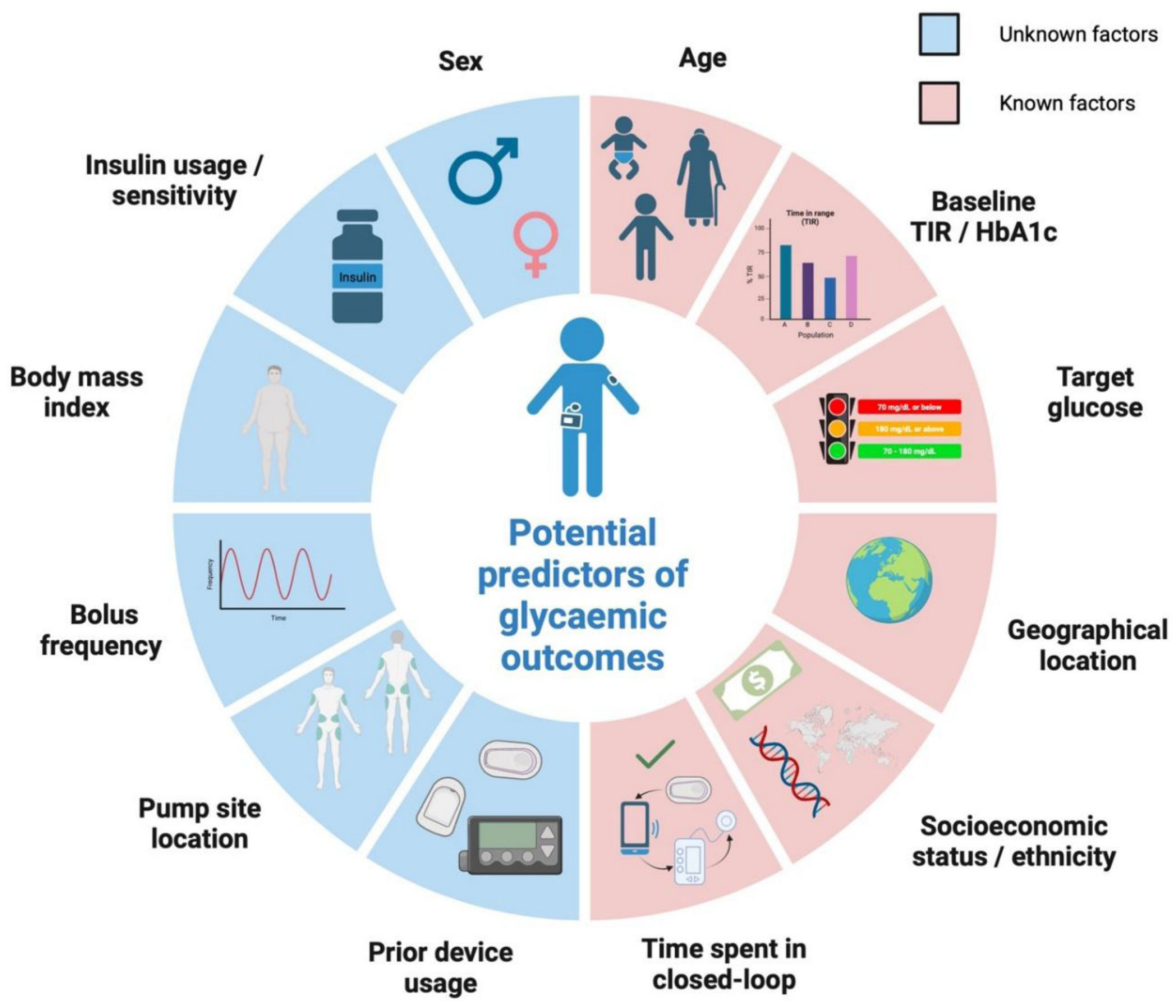
Potential predictors of glycemic outcomes with closed-loop therapy (created in BioRender.com. Boughton, C. (2025) https://BioRender.com/y71f165)

We reviewed the current literature to explore various possibilities, but most factors require further research to confirm their predictive capabilities. These factors are grouped into two categories: known (with a proven impact on glucose outcomes) and unknown (with potential impact or conflicting evidence). This is shown in Figure 3.

### 4.1 Known factors

#### 4.1.1 Age

According to data from the US T1D Exchange registry, mean HbA1c remains stable until 7 years old, increases until the age of 18 and then decreases as age increases. By 25 years old, mean HbA1c is below that of young children. Data from the German/Austrian DPV registry shows a similar pattern, but mean HbA1c is lower across most ages compared to US counterparts [6]. Clinical trials and real-world analyses with various closed-loop systems also show this pattern; children and adolescents with T1D tend to have a lower TIR compared to adults and the elderly. Breton et al. found that TIR was lower for children and adolescents compared to adults and the elderly, both at baseline and when using Control-IQ (Figure 4) [63]. Similarly, real-world evidence from CamAPS FX users showed the same pattern (Figure 5) [58]. In a randomized control trial of AndroidAPS (a DIY open source closed-loop system), children had a TIR of 68% (increased from a baseline of 57%), while adults had a TIR of 75% (increased from 65% at baseline) [39]. With Omnipod 5, children (6-13 years old) had a TIR of 68% compared to 74% in 14-70 year olds [44]. Real-world data with Omnipod 5 showed a similar pattern; TIR decreased from very young children to adolescence and then increased with older age [62].

However, there is likely some selection bias of individuals using closed-loop therapy per age group due to differences in reimbursement policies. For example, in the UK all children and young people are eligible for closed-loop therapy, but is only reimbursed for adults who fulfil certain criteria. Older adults using closed-loop therapy are likely to be those with high health literacy and engagement which may also influence this trend.

When assessing the magnitude of increase in TIR with closed-loop systems rather than just the TIR, some studies show that this is similar across the age groups [39, 63], while one study using Omnipod 5 showed a larger effect size in children but they had a higher baseline HbA1c [44]. Additionally, the treatment effect in a meta-analysis of the Control-IQ system showed that children and young adults had a larger improvement in TIR than adults; TIR improved by the most for 14-25 year olds, followed by <6 year olds, 6-14 year olds, 25-50 year olds and finally those 50 years old or older [66]. However, due to potential differences in baseline TIR / HbA1c it is difficult to determine whether the differences in effect size by age are due to age related factors or baseline TIR / HbA1c.

The data suggests that TIR improves with increasing age, except for a dip during adolescence. This pattern appears consistent for individuals with and without closed-loop systems. However, the magnitude of improvement from using a closed-loop system is either slightly higher in children (who have lower baseline TIR or higher baseline HbA1c) than adults or similar across age groups. It remains unclear whether these closed-loop outcomes are influenced by baseline TIR (which differs by age group), by selection bias regarding who uses the systems within each age group, or by other age-related factors.

#### 4.1.2 Baseline TIR / HbA1c

Baseline TIR has been shown to correlate with TIR during closed-loop usage, with higher baseline TIR associated with higher TIR with closed-loop [42, 67, 68]. This was also seen in 4.1.1 above where age groups with a lower baseline TIR had a lower TIR during closed-loop therapy. Other studies have suggested that baseline HbA1c also influences TIR with closed-loop; individuals with a lower baseline HbA1c tend to reach a higher TIR when using a closed-loop system [42, 59, 64, 69].

A meta-analysis of Control-IQ trial data showed statistically significant interaction between improvement in TIR and baseline HbA1c and improvement in TIR and baseline TIR; improvement in TIR increased as HbA1c increased, and decreased as baseline TIR increased [66]. Other studies showed that improvements in TIR were either similar [39, 63], or of greater magnitude in individuals starting with a lower baseline TIR [42, 44, 67]. Similarly, individuals with a higher HbA1c experienced a larger increase in TIR [42, 59] and a greater decrease in HbA1c with closed-loop therapy [40]. However, individuals with higher baseline TIR / lower baseline HbA1c still achieve a higher TIR compared to those starting with a lower baseline TIR or higher HbA1c.

#### 4.1.3 Target glucose

Closed-loop system users applying a lower algorithm glucose target had a higher TIR when using Omnipod 5. TBR was highest at the lowest target and lowest at the highest target, however mean TBR across all targets was below 2% which is below the 4% recommended by the ADA and EASD [8, 62]. When using the target of 110mg/dL compared to higher values of 120mg/dL or between 130 and 150mg/dL, the highest proportion of users (40%) met all ADA / EASD guidelines for TBR, TIR and TAR [8]. Studies with the Medtronic 780G closed-loop system also showed that a lower target (100mg/dL) was correlated with the highest TIR [68, 70].

#### 4.1.4 Geographical location

People with T1D appear to have different outcomes across different countries. Studies with closed-loop systems that break down outcomes by geographical location often lack baseline data, making it difficult to determine whether better outcomes are due to differences in baseline TIR / HbA1c (as discussed in 4.1.2) [58, 60]. In a real-world analysis of Medtronic 780G users, only 42% of users in Qatar achieved >70% TIR as recommended by the ADA while Romania had 89% of users. Romania had the highest average TIR of 80% while Qatar had the lowest at 67% [60, 71]. However, differences in reimbursement policies and the relatively small number of users in these countries make these differences difficult to interpret. In a study using CamAPS FX, mean TIR was 9 percentage points higher in Australia and Austria than in the UK [58]. It is important to assess whether these differences are due to response to the closed-loop system or are purely due to differences in baseline TIR / HbA1c.

#### 4.1.5 Socioeconomic status / ethnicity

It is documented that people with T1D from an ethnic minority background have sub-optimal glucose outcomes (higher HbA1c and/or lower time in range) [6, 29]. However, a meta-analysis of studies with the Control-IQ closed-loop system shows there is no statistically significant difference in improvement in glycemic control across different ethnicities or socioeconomic status [66]. While data is limited to specific systems, this suggests that documented differences in glucose outcomes are more likely due to access to technology rather than performance of the closed-loop systems. Disparities in access based on both socioeconomic status and ethnicity are documented [72–74] and need to be addressed.

#### 4.1.6 Time spent in closed-loop

When less time is spent in closed-loop mode, the benefits of the system cannot be experienced. Studies have shown a strong positive correlation between time spent in “auto-mode” and glycemic outcomes when using the Medtronic 670G closed-loop system and prototypes of the Cambridge closed-loop system; a higher time in auto-mode was associated with increased TIR and lower HbA1c [75, 76]. This highlights the importance of usability of closed-loop systems for developers.

### 4.2 Unknown factors

#### 4.2.1 Sex

There does not seem to be any difference in glycemic outcomes with closed-loop systems between females and males [42, 64]. However device usage [77, 78] and insulin requirements [79] vary between the sexes.

Females with T1D face additional complicating factors, including menstrual cycles, pregnancy and menopause. Insulin requirements can vary during different phases of the menstrual cycle which makes glycemic control more challenging [80, 81]. Mesa et al. showed that a closed-loop system reduced TBR and increased TIR in all phases compared to sensor augmented pump therapy [81], which highlights the potential of these systems for those who struggle with glucose control due to the menstrual cycle.

More research is needed to fully understand whether there is a clinically relevant difference in glycemic outcomes with closed-loop between female and male patients with T1D at different life-stages as currently available evidence is conflicting [82, 83].

#### 4.2.2 Prior device usage

There are varied outcomes when assessing the impact of prior device usage on closed-loop outcomes. Studies show that there is no statistically significant difference in effect size between those using insulin pump therapy versus MDI and CGM vs no CGM before starting Control-IQ [66] or Medtronic 670G closed-loop therapy [84].

#### 4.2.3 Pump site location

Certain insulin pump site locations have more consistent or faster absorption (for example the abdomen) than other sites (for example legs, buttocks, or arms). Slower absorption can increase glucose fluctuations and impact the efficacy of closed-loop systems, postprandially when trying to bring hyperglycemia back into range. Hypoglycemia may also increase if pump sites are near muscles during exercise or if absorption is faster than normal [85, 86]. However, to our knowledge there are no studies investigating the influence of pump site location on closed-loop performance.

#### 4.2.4 Bolus frequency

Bolus frequency seems to influence TIR with closed-loop therapy; a higher bolus frequency and/or a higher proportion of manual bolus insulin delivery results in improved glycemic control. While one study showed that there was no association [67], others found that increased frequency of bolusing and a higher proportion of insulin delivery via bolus was correlated with a higher TIR [87]; the median TIR in individuals who bolused <4 times per day was 59.9% compared to 72.4% amongst those who bolused 4 or more times per day [62].

#### 4.2.5 Body Mass Index (BMI)

When assessing the association between BMI and closed-loop outcomes one study using Control-IQ reported that those with a BMI of >25 had a slightly higher TIR than those with a BMI of ≤25 but this was not statistically significant [42]. Another study using the DIY Loop system reported no difference in TIR between users across three categories of BMI – underweight, overweight and obese [64].

There are also further interrelated and complicating factors; those with a higher BMI are likely to have greater insulin resistance [88] and increased insulin doses [89] and may have different dietary intake than those with lower BMI.

#### 4.2.6 Insulin usage / sensitivity

Individuals with lower insulin requirements sometimes have residual C-peptide which may help with maintaining optimal glycemia [90]. It is not clear whether users with lower insulin doses due to either residual C-peptide or higher insulin sensitivity have different outcomes with closed-loop than users with higher insulin doses (those with no residual C-peptide or lower insulin sensitivity).

#### 4.2.7 Other

Other potential predictors also need to be explored in more depth, including comorbidities, other medications and psychosocial factors that may influence glucose outcomes. These factors have the potential to impact engagement with diabetes management and therefore glycemic outcomes.

## 5.0 Barriers / Limitations of closed-loop therapies

### 5.1 Insulin action

The efficacy of hybrid closed-loop systems is limited by the slow subcutaneous absorption of current rapid acting insulin analogues. This leads to increased post-prandial glycemic excursions due to delayed onset and slow offset of insulin action [91]. It also means that announcing and pre-bolusing for meals is still recommended for optimal glycemic outcomes [92]. Ultra-rapid acting insulins such as Fiasp and Lyumjev have been developed with excipients that speed up absorption [93]. While some closed-loop studies have shown small improvements in TIR and/or TBR [94–96], outcomes have been inconsistent [97–99]. This highlights the need to develop even faster-acting insulins to enable further advancements in closed-loop performance, particularly to improve post-prandial outcomes and ultimately achieve fully closed-loop therapy.

### 5.2 Education / training

Education and training are important to ensure safety and efficacy when starting closed-loop therapy. Extra support during the initial stages is important for optimal outcomes, continuation of the closed-loop system and to help with troubleshooting [100, 101]. Training requirements will vary depending on prior device usage. If the person with T1D is new to CGM or insulin pump therapy, thorough training on each component will need to be completed. However, for individuals with prior experience using sensor-augmented pump therapy, training can be focused on the details of the specific automated insulin delivery system [5]. More comprehensive initialization and closed-loop management recommendations can be found in the Consensus Recommendations by Philip et al. [5].

Importantly, training of closed-loop system users requires healthcare professionals to be trained first. This time commitment, combined with the required troubleshooting, optimizing of settings and follow up can make the burden on healthcare professionals high [101, 102]. However, this is acknowledged and there is an effort to address this [103]. Training can be done in person but can also be completed via videoconference, with extra support from videos, e-learning, or simulation apps. This can help to reduce the burden and time commitment for healthcare providers while also ensuring the most effective learning experience for users [104–107]. Different training approaches allows individuals with different education and health literacy backgrounds better access the technology.

### 5.3 Access

Hybrid closed-loop insulin delivery systems have become the standard of clinical care to manage T1D [5]. In the UK the National Institute for Health and Care Excellence (NICE) have recommended hybrid closed-loop systems for all children, young people, pregnant women. It is also recommended for adults meeting certain criteria [108]. However, access to closed-loop systems can be varied and the proportion of people with T1D using insulin pumps, CGMs and automated insulin delivery systems varies by age, geographical region and ethnic group [7, 109–111]. Disparities in access to diabetes technology for those from disadvantaged areas as well as ethnic minorities have been widely documented [72, 73, 109]. Despite the ability of closed-loop systems to reduce complications and improve quality of life compared to other management methods and a growing body of evidence suggesting that closed-loop systems are cost-effective [112–114], in some healthcare systems, this advanced technology is not covered by the state, and requires private insurance, which can lead to inequitable access. The variation in infrastructure and available resources also impacts training of healthcare professionals and hence implementation of hybrid closed-loop therapies in certain areas.

Diabetes management requires a high burden of self-care. This means individuals with learning disabilities, visual or hearing impairments or dexterity problems may encounter difficulties with some aspects of care [115]. There is a growing number of case reports highlighting benefits of CGMs and insulin pump therapy in these populations [116, 117]. However, there are some limitations as insulin pumps are still not particularly accessible to visually impaired individuals [118], some people with autism spectrum disorder may struggle with the additional devices due to sensory issues [119] and inserting devices with limited dexterity can be difficult. In some circumstances, such as limited dexterity, other diabetes management techniques are just as difficult. Technological advancements and closed-loop systems have the potential to address some of these issues and reduce the burden of care. It is also important to ensure that training programs and materials are made accessible to individuals with different learning requirements. Closed-loop therapies have the potential to benefit all individuals with T1D, making it crucial to address existing disparities in access and ensure that both the systems and training are accessible to all.

### 5.4 Other issues

To achieve optimal outcomes, hybrid closed-loop systems require all components to function safely and effectively. Issues like CGM inaccuracies or failures, insulin pump site problems, or device communication errors can impact the ability to deliver insulin or make accurate predictions thereby disrupting the system’s performance. The lag in CGM data can delay responses to glucose fluctuations, and systems may struggle with unpredictable activities like exercise unless the appropriate setting is activated.

Additionally, accurate carbohydrate counting remains challenging, which can impact on outcomes.

## 6.0 Future of closed loop therapies

### 6.1 Fully closed-loop

Currently available hybrid closed-loop systems still require bolusing for meals or meal announcements. Fully closed-loop therapies aim to alleviate the need for this which has the potential to reduce the burden of diabetes self-care and widen access while also reducing the workload burden for healthcare professionals. Fully closed-loop systems have been shown to improve quality of life and glucose control in certain populations compared to sensor-augmented pump therapy [120].

CamAPS HX (CamDiab), a fully closed-loop system improved glucose outcomes in adults with T1D with suboptimal HbA1c at baseline (≥8.0% / ≥64mmol/mol). TIR increased by 13 percentage points to a mean of 50% [121]. The question arises whether a fully closed-loop system would allow a greater improvement or a similar improvement to a higher mean TIR if baseline TIR was higher or HbA1c lower. Potential predictors of glycemic outcomes also need to be investigated to assess whether algorithms could be further optimized to improve performance. Other fully closed-loop feasibility studies also show that the diabetes management burden is reduced, but postprandial hyperglycemia is relatively common so glycemic control is compromised [122]. It would be interesting to investigate why some people respond better to the system than others when variables such as bolus frequency and carbohydrate counting accuracy are removed from the equation.

### 6.2 Adjunctive therapies / faster acting insulins

Fully and hybrid closed-loop systems are limited by the delay in insulin absorption via subcutaneous delivery. The slow absorption has a particular effect on postprandial glucose control, especially in fully closed-loop systems where meals are not announced so pre-bolusing does not occur [122, 123]. This challenge could be addressed by developing quicker rapid-acting insulin analogues with time-action profiles that more closely mimic physiological responses or by exploring alternative routes of administration to improve absorption time. Inhaled insulin [124] and intraperitoneal insulin delivery are currently under investigation [125, 126].

Bi-hormonal closed-loop systems with glucagon and pramlintide are also being investigated. Incorporating glucagon offers the potential for algorithms to be more aggressive, which could lead to improved glucose control. Additionally, exercise-induced hypoglycemia can be challenging to prevent solely by reducing insulin doses [123], but adding glucagon has shown potential. Van Bon et al. showed a mean TIR of 80% when adults with T1D used a fully closed-loop system with both insulin and glucagon over a period of 12 months (no comparator therapy) [127], while the iLet bi-hormonal system increased TIR compared to the single hormone (insulin-only) system (79% vs. 72%) and decreased TBR (2% vs. 4%) [128]. Some side-effects of the glucagon such as nausea and reactions at the infusion site were reported [127, 128]. An additional burden that must be considered is the requirement to wear two pumps and two infusion sets.

Pramlintide (a synthetic amylin analogue) is being tested in multi-hormone closed loop therapy [129–131] Pramlintide reduces postprandial excursions [131], and increased TIR from 74% to 84% when used in a dual-hormone artificial pancreas system with rapid-acting insulin [129]. Pramlintide can cause gastrointestinal side-effects impacting on tolerability.

Another option is using other adjunctive medications such as glucagon-like peptide 1 receptor agonists (GLP-1) [131, 132] or sodium-glucose cotransporter 2 (SGLT-2) inhibitors [133, 134] in conjunction with closed-loop systems. GLP-1 receptor agonists delay gastric emptying, suppress glucagon secretion, and can reduce postprandial hyperglycemia, offering the potential to further optimize glucose control beyond what insulin-only closed-loop systems can achieve. Dapagliflozin increased TIR by an average of 259 minutes per day compared to placebo when used as an adjunct to fully closed-loop therapy [133]. However, adjunctive Empagliflozin was not able to eliminate the need for carbohydrate counting or meal announcements, showing inferiority when compared to a hybrid closed-loop approach [134]. GLP-1 and SGLT-2 therapies have the potential to increase risk of diabetic ketoacidosis so symptoms must be carefully monitored.

Further information about adjunctive therapies with closed-loop therapy can be found in the review by Srinivasan et al. [135].

### 6.3 Extended wear infusion sets / CGMs

Most infusion sets are currently required to be changed every 48 to 72 hours which adds an additional burden to users. CGMs have a longer duration of wear of between 7 and 14 days. Extended wear infusion sets have recently been developed; and users have expressed increased satisfaction when using them. These infusion sets offer a reduction in device burden which is a potential barrier to closed-loop system use. There is currently one approved extended wear infusion set, which is associated with stable glycemic outcomes throughout the wear duration [136, 137].

### 6.4 Interoperability

Different closed-loop systems are used with different insulin pumps and CGMs. They have different algorithms, customizable targets, infusion sets, and some have the ability to interact with the system using a mobile device. Individuals with T1D have different requirements and preferences. The development of interoperable systems would provide users with increased flexibility, potentially improving comfort, quality of life, adherence and glycemic outcomes. But this does rely on complex agreements between device manufacturers which can be prohibitive

## 7.0 Conclusion

Hybrid closed-loop systems have emerged as the standard of care for managing T1D, primarily due to their improvements in glycemic outcomes and quality of life. Both clinical trials and real-world studies validate the safety and efficacy of the systems that are currently in clinical use. They demonstrate unique benefits across different age groups but are safe and effective in all populations. There are various factors that may influence glycemic outcomes in individuals using closed-loop systems, for example baseline HbA1c or TIR, age, geographical location, ethnicity, insulin sensitivity or BMI. Further research is needed to clarify whether these factors serve as direct predictors of glycemic outcomes or merely correlate with other factors such as baseline characteristics.

Despite the advancements, certain limitations persist, such as the slow absorption rates of current rapid-acting insulins, the training required for effective device utilization, and challenges related to access of the technology. The future of diabetes management lies in the evolution of fully closed-loop systems which may need to be paired with adjunctive medications and enhanced insulin delivery methods that facilitate faster absorption. This approach aims to alleviate user burden while further improving glycemic outcomes. Ongoing research into potential predictors of diabetes management outcomes with closed-loop is important, as identifying these factors will enable the refinement of algorithms or appropriate interventions to improve outcomes globally.

## 8.0 Expert Opinion

Hybrid closed-loop systems are becoming increasingly common in the treatment of T1D, as their benefits become clearer, and their safety and efficacy are well-established. A wide range of hybrid closed-loop systems are commercially available, with new component devices and systems under development and existing systems being advanced. These systems have demonstrated positive outcomes and safety in both clinical trials and real-world data. This extends across all age groups. The variety of available options is crucial, as different algorithms may be more suitable for different individuals. Moreover, these systems use different insulin pumps and cannulas and continuous glucose monitors (CGMs) which cater to individual preferences. For instance, athletes may prefer waterproof, tubeless insulin pumps, while other users may require a steel cannula, which are available only in tubed insulin pumps. The availability of choice hopefully allows improved glycemic outcomes, enhanced user engagement, improves comfort and contributes to better quality of life. Improving interoperability between devices would allow for even greater customization for users.

### 8.1 Limitations to implementation

There are significant disparities in access to closed-loop systems at present based on factors such as geographical location, socioeconomic status, and ethnicity. Reimbursement policies often limit access to this advanced technology. The training required for both healthcare providers and users is time-consuming and places a substantial burden on medical staff to be familiar with the different systems. Additionally, individuals with disabilities, such as visual impairments or autism, may require tailored educational methods. The financial cost of these systems, combined with the training needed, adds a barrier to their widespread clinical adoption. However, the benefits of improved glycemic outcomes, better quality of life, and the potential for reduced complications outweigh the costs in health economic analyses. To ensure all people with T1D can access this life-changing technology, it is important to update educational approaches and expand reimbursement policies.

### 8.2 Future developments

While closed-loop systems have been shown to improve glycemic control and reduce the burden of care, individual users experience varying glucose outcomes. Identifying potential predictors of glycemic outcomes could enable targeted interventions, such as adjustments in clinical guidelines, education, support, or algorithm modifications. The goal is to further optimize closed-loop systems, to help more individuals achieve optimal outcomes.

Currently, available closed-loop systems are hybrid, meaning bolusing for meals or announcing meals is still required. While fully closed-loop systems are under development, all systems are limited by the slow subcutaneous absorption of rapid-acting insulins, which can cause postprandial spikes and increase glucose fluctuations. Research into faster-acting insulins or alternative methods of insulin delivery to provide more physiologically accurate time-action profiles is therefore crucial. Additionally, the use of bihormonal approaches with glucagon or pramlintide or adjunctive therapies such as glucagon-like peptide 1 (GLP-1), and sodium-glucose cotransporter 2 (SGLT-2) inhibitors is being explored. Fully closed-loop systems aim to minimize user input, reducing the burden of care for both users and healthcare providers. However, eliminating the need to bolus for meals should not compromise glycemic control.

Promising developments in T1D research, such as islet cell transplants and stem cell therapy, hold potential for a cure. While a cure is the ultimate solution, the future of diabetes management at present lies in advancing closed-loop systems. The ultimate goal is to create a system that requires no user involvement while still achieving optimal glycemic outcomes.

Closed-loop systems have rapidly evolved over the past decade, and current research indicates that further advancements are likely. While the short-term clinical and psychological benefits of these systems have been demonstrated, it is crucial to continue assessing their impact, especially in reducing long-term complications associated with T1D. Efforts to improve access are underway, and it is likely that the clinical implementation of this technology will continue to grow.

## Figures and Tables

**Figure 1:**
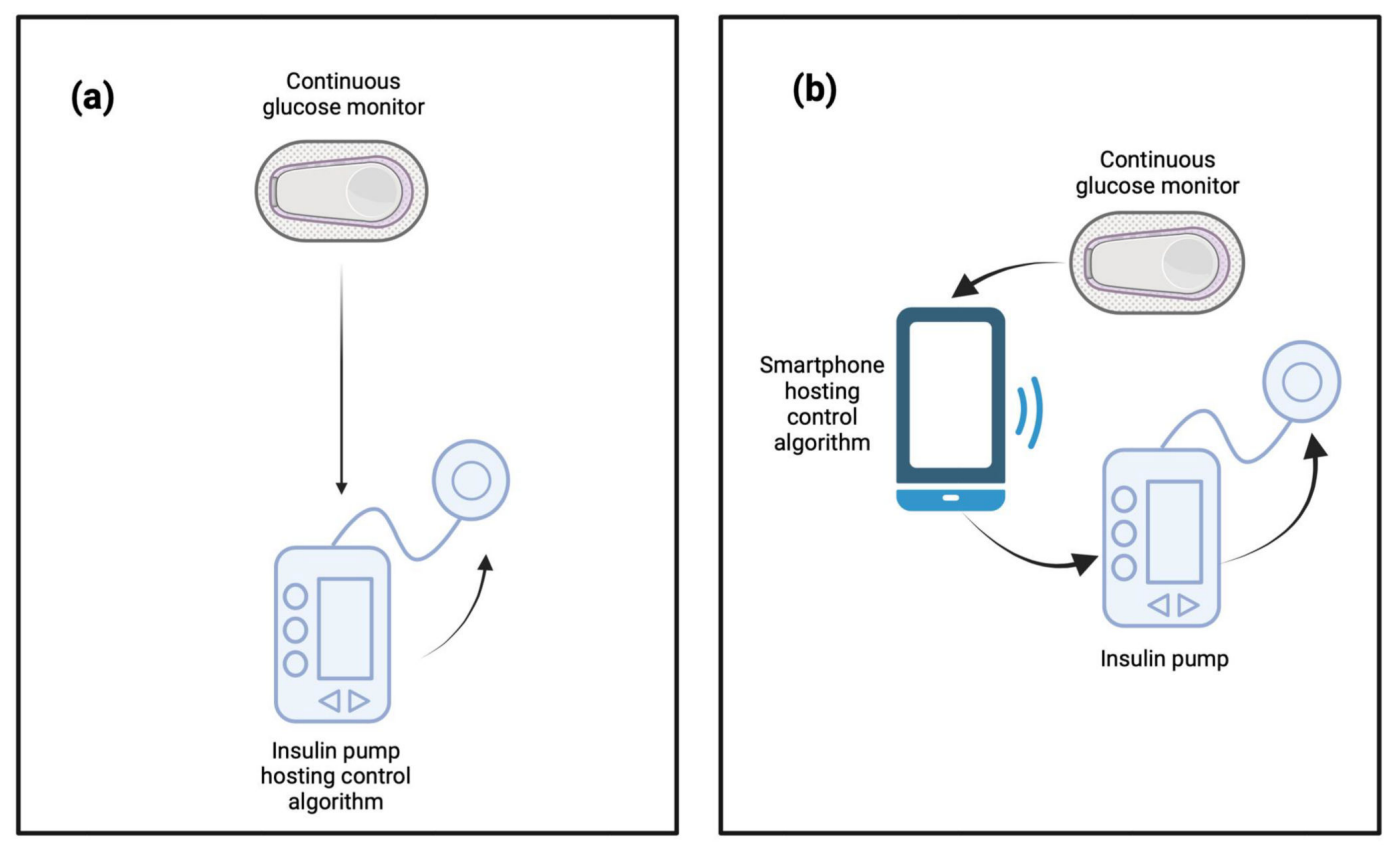
Generic closed loop figure in systems where the algorithm is housed on (a) the insulin pump (b) a compatible mobile device (created in BioRender.com. Boughton, C. (2025) https://BioRender.com/h83p169)

**Table 1:** Randomized clinical trials for currently available AID systems

Age (sample size)	AID system (author)	Study design (type, duration, comparison group)	Study location	Glycemic outcomes			
				TIR (70 – 180 mg/dL)	Δ TIR	TBR (<70 mg/dL)	Δ TBR
**Young Children**							
Age 1 – 7 yrs (N=74)	CamAPS FX (Ware et al. [22])	Randomized crossover trial, 2x 16 week periods, SAP therapy	Austria, Germany, Luxembourg, United Kingdom	Control = 63% ^a^ AID = 72% ^a^	9 (7 to 10)^†^	Control = 4.5% ^b^ AID = 4.9% ^b^	0.1 (-0.4 to 0.5)^†^
Age 2 – 6 yrs (Control: N=34) (AID: N=68)	Tandem CIQ (Wadwa et al. [23])	Randomized parallel trial, 26 weeks, SAP or MDI therapy	United States	Control baseline = 55% ^a^ Control = 56% ^a^ AID Baseline = 57% ^a^ AID = 69% ^a^	12 (10 to 15)^†^	Control baseline = 2.7%^a^ Control = 3.0% ^a^ AID baseline = 3.0% ^a^ AID = 3.0% ^a^	−0.2 (−0.7 to 0.4)^†^
**Adolescents / Young Adults**							
Age 6 – 18 yrs (Control: N=62) (AID: N=57)	CamAPS FX (Ware et al. [37])	Randomized parallel trial, 26 weeks, insulin pump therapy ± CGM	United Kingdom, United States	Control baseline = 46% ^a^ Control = 47% ^a^ AID Control = 47% ^a^ AID = 54% ^a^	7 (2 to 11)^†^	Control baseline = 4.9% ^b^ Control = 5.4% ^b^ AID Control = 6.1% ^b^ AID = 6.1% ^b^	0.5 (−1.8 to 2.8)^†^
Age 7 – 15 yrs (Control: N=27) (AID: N=21)	Android APS (DIY) (Burnside et al. [39])	Randomized parallel trial, 24 weeks, SAP therapy	New Zealand	Control baseline = 55% ^a^ Control = 53% ^a^ AID baseline = 57% ^a^ AID = 68% ^a^	13 (6 to 20)^†^	Control baseline = 3.7% ^a^ Control = 2.7% ^a^ AID baseline = 3.5% ^a^ AID = 2.1% ^a^	-0.5 (−1.6 to 0.5)^†^
Age 14 – 29 yrs (N=113)	Medtronic 780G (Bergenstal et al. [43])	Randomized crossover trial, 2 × 12 weeks, Medtronic 670G	United States, Germany, Israel, Slovenia	Baseline = 57% ^a^ Control (670G) = 63% ^a^ AID (780G) = 67% ^a^	NA	Baseline = 2.3% ^a^ Control (670G) = 2.1% ^a^ AID (780G) = 2.1% ^a^	NA
**Children / Adolescents / Adults**							
Age 6 – 79 yrs (Control: N=107) (AID: N=219)	iLet (Bionic Pancreas Research Group [40])	Randomized parallel trial, 13 weeks, SAP or MDI therapy	United States	Control baseline =51% ^b^ Control = 54% ^b^ AID baseline = 51% ^b^ AID = 65% ^b^	11 (9 to 13) ^†^	Control baseline = 1.4% ^a^ Control = 1.8% ^a^ AID baseline = 1.5% ^a^ AID = 1.8% ^a^	−0.1 (−0.3 to 0.2) ^†^
Age 7 – 80 yrs (N=59)	Medtronic 780G (Collyns et al. [41])	Randomized crossover trial, 2x 4 week periods, SAP with PLGS	New Zealand	Control = 58% ^a^ AID = 70% ^a^	13 ± 9 §	Control = 2.5% ^a^ AID = 2.1% ^a^	-0.4 ± 1.3 §
Age 14 – 71 yrs (Control: N=56) (AID: N=112)	Tandem CIQ (Brown et al. [42])	Randomized parallel trial, 26 weeks, SAP therapy	United States	Control baseline = 59% ^a^ Control = 59% ^a^ AID baseline = 61% ^a^ AID = 71% ^a^	11 (9 to 14) ^¶^	Control baseline = 2.8% ^a^ Control = 2.3% ^a^ AID baseline = 3.6% ^a^ AID = 1.6% ^a^	−0.9 (−1.2 to −0.6) ^¶^
Age 18 – 70 yrs (Control: N=62) (AID: N=131)	Omnipod 5 (Renard et al. [52])	Randomized parallel trial, 13 weeks, SAP therapy	United States France	Control baseline = 41% ^a^ Control = 44% ^a^ AID baseline = 44% ^a^ AID = 61% ^a^	18 (14 to 21) ^†^	Control baseline =1.7% ^a^ Control = 1.8% ^a^ AID baseline = 1.7% ^a^ AID = 1.2% ^a^	−0.4 (−0.6 to −0.1) ^†^
Age ≥6 yrs (Control: N=40) (AID: N=46)	CampAPS FX (Tauschmann et al. [53])	Randomized parallel trial, 12 weeks, SAP therapy	United Kingdom, United States	Control baseline = 52% ^a^ Control = 54% ^a^ AID baseline = 52% ^a^ AID = 65% ^a^	11 (8 to 14) ^†^	Control baseline = 3.3% ^b^ Control = 3.9% ^b^ AID baseline = 3.5% ^b^ AID = 2.6% ^b^	−0.8 (−1.4 to −0.2) ^†^
Age ≥18 yrs (N=63)	Diabeloop DBLG1 (Benhamou et al. [51])	Randomized crossover trial, 2x 12 week periods, SAP therapy	France	Control = 59% ^a^ AID = 69% ^a^	9 (6 to 12) ^‡^	Control = 4.3% ^a^ AID = 2.0% ^a^	−2.4 (−3.0 to −1.7) ^‡^
Age 16 – 70 yrs (Control: N=26) (AID: N=22)	AndroidAPS (DIY) (Burnside et al. [39])	Randomized parallel trial, 26 weeks, SAP therapy	New Zealand	Control baseline = 60% ^a^ Control = 57% ^a^ AID baseline = 65% ^a^ AID = 75% ^a^	15 (9 to 22) ^†^	Control baseline = 1.7% ^a^ Control = 1.8% ^a^ AID baseline = 2.3% ^a^ AID = 1.6% ^a^	0.4 (−1.4 to 0.6) ^†^

Abbreviations: AID: automated insulin delivery system; CGM: continuous glucose sensor; MDI: multiple daily injections; NA: not available; PLGS: predictive low glucose suspend; SAP: sensor-augmented pump; TBR: time below range; TIR: time in range; yrs: years old

a mean

b median

* sample size is those who completed the trial period

† mean adjusted difference (95% CI)

‡ paired difference

§ Absolute difference

¶ risk adjusted difference

**Table 2:** Single arm clinical trials for currently used AID systems that do not have randomized trials

Age (sample size)	AID system (author)	Study design (type, duration, comparison group)	Study location	Glycaemic outcomes	
				TIR (70 – 180 mg/dL)	TBR (<70 mg/dL)
**Young Children**					
Age 2 – 6 yrs (N=35)	Medtronic 780G (Pulkkinen et al. [25])	Single arm trial, 12 weeks, Medtronic 640G, 670G or MDI with CGM	Finland	Baseline = 58.3% ^a^ AID = 66.6% ^a^	Baseline = 3.0% ^a^ AID = 3.2% ^a^
Age 2 – 5 yrs (N=80)	Omnipod5 (Sherr et al. [24])	Single arm trial, 13 weeks, standard therapy (97.5% CGM 85% insulin pump 15% MDI)	United States	Baseline = 57.2% ^a^ AID = 68.1% ^a^	Baseline = 3.4% ^a^ AID = 2.5% ^a^
**Children / Adolescents**					
Age 6 – 13 yrs (N=112)	Omnipod5 (Brown et al. [44])	Single arm trial, 13 weeks, standard therapy (96% CGM 89% insulin pump, 12% MDI)	United States	Baseline = 52.5% ^a^ AID = 68.0% ^a^	Baseline = 2.2% ^a^ AID = 1.8% ^a^

Abbreviations: AID: automated insulin delivery system; CGM: continuous glucose sensor; MDI: multiple daily injections; TBR: time below range; TIR: time in range; yrs: years old

a mean,

b median

**Table 3:** Real-world studies for currently used AID systems

	Author	Study design (type, duration)	Study population (age, number of participants)	Study location	Glycaemic outcomes		
					Baseline*	TIR	TBR
**CamAPS FX**	Alwan et al. [58]	7 month retrospective real- world data analysis	Age ≥1 yrs, N = 1,085	Australia, Austria, Czech Republic, Denmark, Finland, Germany, Ireland, Italy, Luxembourg, Netherlands, Poland, Spain, Sweden, Switzerland, United Kingdom	NA	73% ^a^	2.3% ^b^
**Diabeloop DBLG1**	Benhamou et al. [59]	12 month retrospective real- world data analysis	Age ≥18 yrs, N = 3,706	Germany	NA	72% ^b^	0.9% ^b^
**Loop (DIY)**	Lum et al. [64]	6 months real-world prospective study	Age 1 – 71 yrs, N = 558	United States	TIR = 67%^b^ TBR = 3%^a^	73% ^a^	2.8% ^b^
**Medtronic 780G**	Choudhary et al. [60]	3 year retrospective CareLink system data analysis	Age ≥2 yrs, N = 101,629	Europe, Middle East, Africa	NA	72% ^a^	2% ^a^
**Omnipod 5**	Forlenza et al. [62]	12 month retrospective real- world data analysis	Age 2 - 17 yrs, N = 22,162	United States	NA	61% ^b^	1.2% ^b^
			Age ≥18 yrs, N = 47,740	United States	NA	66% ^b^	0.9% ^b^
**Tandem CIQ**	Breton et al. [63]	12 month retrospective Tandem t:connect system data analysis	Age ≥6 yrs N = 9,451	United States	TIR = 64%^a ^TBR = ~1% ^a^	74% ^b^	~1% ^b^

Abbreviations: NA: not applicable, yrs: years old

a mean,

b median

* not all individuals had baseline data

**Table 4:** Clinically used automated insulin delivery systems

	CamAPS FX	Diabeloop DBLG1	iLet	Medtronic 780G	Omnipod 5	Tandem CIQ
**Compatible insulin pump**	YpsoPump, Dana RS or I pump	Kaleido	iLet pump	Minimed 780G	Omnipod 5 patch pump	tslim:X2 or mobi (US only)
**Compatible CGM**	Dexcom G6, Freestyle Libre 3	Dexcom G6	Dexcom G6/G7	Guardian 3 (requires calibration)/Guardian 4	Dexcom G6, FreeStyle Libre 2 Plus	Dexcom G6 / G7, Freestyle Libre 2 Plus
**Algorithm**	MPC adaptive algorithm, calculates insulin sensitivity, active insulin time and carbohydrate bioavailability	MPC inspired - physiological framework with machine learning (short and long term learning algorithms as well as meal management algorithm for meal boluses)	3 algorithms (basal insulin, bolus correction and meal aware algorithm)	PID algorithm with insulin feedback	MPC algorithm, Calculates basal insulin rate based on recent total daily insulin dose	MPC algorithm with pre programmed basal rates and insulin sensitivity factors
**Settings required for initialization**	TDD, weight	TDD, weight, average carbs for standard breakfast, lunch and dinner meal, basal rates (if manual mode)	Weight	TDD, weight, ISF, ICR, basal rate	TDD, ICR, ISF, basal rate	TDD, weight, ISF, ICR, basal rate
**Adaptive learning**	Yes – overall, diurnal and meals	Yes – short and long term	Yes – overall and meal announcement algorithm	Yes - overall	Yes – basal rate for each pod (estimates TDD by multiplying programmed basal insulin by 2)	No
**Autocorrections**	No (algorithm tuned so it is not needed)	Yes	Yes	Yes	No uses basal rates	Yes
**Mode to reduce insulin delivery**	“Ease-off’ target increases by 40 mg/dL, insulin sensitivity increased, programmable duration (10 min to 24 hours or pre-planned)	“Activity mode” increases target by 70 mg/dL, adjustable duration (0 to 24 hours) “Zen mode” increases target by 10-40 mg/dL, adjustable duration (3-8 hours)	No	Temp target at 150 mg/dL, programmable duration	“HypoProtect” target of 150 mg/dL, Basal/bolus/corrections are 50% programmable duration (1 – 72 hours)	Temp target at 140–160 mg/dL, Basal stopped at 80 mg/dL, Manually switch off
**Mode to increase insulin delivery**	“Boost” increases the amount of insulin delivered by assuming increased insulin requirements in a glucose responsive manner, programmable duration	“Aggressiveness” 59% to 174% (hyperglycemia) 50% to 200% (meals) 43% to 186% (normoglycemia)	No	No	No	No
**Target glucose level**	Adjustable between 80–200 mg/dL	Adjustable between 100 – 130 mg/dL	110, 120 or 130 mg/dL	100, 110 or 120 mg/dL	Adjustable between 110 – 150 mg/dL in up to 8 segments throughout the day	Range of 112.5 - 160 mg/dL, or sleep target range of 112.5 - 120 mg/dL
License*	Diabetes requiring insulin, age ≥1 yrs including pregnancy (FDA and CE mark)	T1D, age ≥18 yrs (CE mark)	T1D, ≥6 yrs (FDA)	T1D, age ≥7 yrs (FDA and CE mark)	T1D, age ≥2 (FDA and CE mark)	Diabetes requiring insulin, age ≥2 (FDA and CE mark)
**Remote monitoring**	Yes – CamAPS Companion app	No	CGM only (Dexcom follow)	Yes – Carelink Connect app	Yes – Omnipod VIEW or CGM via Dexcom follow	CGM only (Dexcom follow)
**Bolus from phone**	Yes	No	No	No	Yes	Yes (US only)

Abbreviations: CGM: continuous glucose monitor; CIQ: control IQ; ICR: insulin to carb ratio; ISF: insulin sensitivity factor; MPC: Model Predictive Control; PID: proportional integral derivative; TDD: total daily dose

* Only FDA and CE marking have been included in this table but several hybrid closed-loop systems have also been approved by other regulatory bodies including the Therapeutic Goods Administration (Australia), MEDSAFE (New Zealand) and Medical Devices Directorate (Canada).

## Box 1 Article Highlights

- Hybrid closed-loop systems have become the standard of care to manage type 1 diabetes.
- There are several different closed-loop systems in clinical use across all age groups, offering people with type 1 diabetes the flexibility to choose the system that best suits their needs.
- These systems have been shown to improve glycemic control without increasing the risk of hypoglycemia, while also enhancing quality of life, as demonstrated in both clinical trials and real-world data analyses.
- The slow subcutaneous absorption of current rapid-acting insulins can impact the efficacy of closed-loop systems, particularly post-prandially. Further limitations to closed-loop therapy include the education / training required for both users and healthcare providers and disparities in access to this advanced technology.
- Advancements are being explored in fully closed-loop therapy including multi-hormone closed-loop systems, adjunctive medications used with automated insulin delivery systems, and faster-acting insulins.
- Glucose outcomes with closed-loop systems are variable between individuals which may be attributable to a number of different factors. If these factors can be identified then further actions can be taken to adjust algorithms, enhance education, or update clinical guidelines, with the ultimate aim of optimizing outcomes.
- There also needs to be a focus on improving access to these systems for all individuals with type 1 diabetes.
